# The Evaluation and Detection of the Chemical Bond Between Silane Coupling Agent and Silver Layer on Alkali Activated Fly Ash

**DOI:** 10.3390/ma17215322

**Published:** 2024-10-31

**Authors:** Ranfang Zuo, Jingyun Chen, Jinder Jow, Yang Dong

**Affiliations:** National Institute of Clean-and-Low-Carbon Energy, Future Science City, Changping District, Beijing 102211, China; jingyun.chen.a@chnenergy.com.cn (J.C.); jinde.zhuo@chnenergy.com.cn (J.J.); yang.dong@chnenergy.com.cn (Y.D.)

**Keywords:** alkali activation, modification, silane coupling agent, chemical bond, plating time

## Abstract

In this study, wettability was employed to evaluate the effect of alkali activation by NaOH on different fly ash (FA) particle sizes. The results indicated that the surface wettability of FA particles with 13.8 μm increased from 0.025 g^2^/s to 0.034 g^2^/s after activation by the NaOH solution, which is suitable for silane modification and electroless plating. X-ray photoelectron spectroscopy (XPS) was used to analyze whether three kinds of silane coupling agents coated on FA surfaces could detect the chemical bonds between silane coupling agents coated on the FA surface and silver layers by shortening the plating time. The XPS results demonstrated that N-Ag coordination bonds can be detected by reducing silver plating time to 2 min for Ag-plated FA modified by N-(2-aminoethyl)-3-aminopropyltrimethoxysilane (KH792). However, there were no chemical bonds detected for Ag-plated FA modified by γ-(2,3-epoxypropoxy)propytrimethoxysilane (KH560) and methyltrimethoxysilane (MTMS), even when the satellite peak of Ag disappeared after plating for 80 s. The SEM showed that Ag particles agglomerated on FA surfaces, and even a bare surface was found after modification by KH560 and MTMS, which further proved no chemical bonds between silver layers and the silane coupling agents.

## 1. Introduction

Fly ash (FA), a by-product of coal combustion power plants, has the advantages of chemical and mineralogical suitability, good workability, and easy availability worldwide and can be used in soil amelioration, the construction industry, the ceramic industry, catalysis, depth separation, zeolite synthesis, the filler industry, etc. [1]. The typical particle size range of FA is 0.5 μm to 300 μm [2,3,4]. The particle size of FA can be altered externally either by classification or milling [5]. The particle size of FA plays a predominant role in its reactivity, microstructure, surface area, and surface activity [5,6]. Interestingly, the different sizes of FA vary considerably in terms of chemistry, mineralogy, and reactivity [7,8,9]. The physical and chemical properties of FA affect their options for re-use. To increase the re-use of FA, our research group has developed a technology to classify FA into different particle sizes with high sphericity. Research has indicated that the spherical particle is one of the most valuable materials found in FA [10]. Spherical FA can be used as filler in rubber due to its low density, high sphericity, and good dispersion [11]. However, original FA has a poor interface-binding ability when FA is added to organic matter. A common method of overcoming this drawback is to modify FA with functional groups to change the degree of binding, whereby alkali activation is necessary before modification. Researchers have used hydrophilic and hydrophobic modifiers to modify FA, which showed specific enhancements in shielding properties [12,13]. FA shows the characteristics of inorganic filler, and surface modification research is very important in the field of coating. Thus, we have employed a modified electroless plating method to prepare Ag-plated FA powders, which use classified spherical FA as a substrate [14]. Furthermore, FA particle surfaces have been activated before using the electroless plating process because of their lack of electrical and magnetic conductivity, and our previous work indicated that alkali activation and silane modification have a synergistic effect on silver plating [14]. At present, there are no literature reports on the binding modes between different kinds of silane coupling agents and silver particles on the FA surface.

In this study, two particle sizes of FA were obtained by our classification technology and used as a substrate for silver plating. Alkali activation and silane modification were employed to activate the FA surface before electroless plating. We studied the effect of the NaOH concentration and silane solution on FA surface activation and modification, respectively. The present paper focused on investigating the bonding form between silver particles and pretreated FA particles via alkali activation and three different kinds of silane coupling agents used for surface modification. The purpose of this study was to explore the suitable coupling agent and characterization method for detecting chemical bonds between silane coupling agents on the FA surface and silver particles plated on the FA surface.

## 2. Experimental

### 2.1. Materials and Chemicals

Spherical fly ash particles (chemical composition (as shown in Table 1): SiO_2_, Al_2_O_3_, CaO, Fe_2_O_3_, K_2_O, TiO_2_, Na_2_O, MgO; particle size: D50 = 2.9, 13.8 μm; density: 2.3–2.5 g/cm^3^; combustion loss (as shown in Table 1): 3.40%, 6.90%) were obtained from fly ash produced by the Sanhe power plant by our classifying technology. SH012F and SH090 represent FA with different particle sizes after classification. The three kinds of silane coupling agents (KH792: N-(2-aminoethyl)-3-aminopropyltrimethoxysilane, MTMS: methyltrimethoxysilane, and KH560: γ-(2,3-epoxypropoxy)propytrimethoxysilane) were supplied by Qufu chemical Co., Ltd. (Shandong, China). Sodium hydroxide (NaOH, AR) was purchased from Sinopharm Group Chemical Reagent Co., Ltd. (China).

### 2.2. Modification

1. Alkali activation: Spherical fly ash (FA) particles were dispersed in different concentrations of a NaOH solution (0.3, 0.6, and 1.2 mol/L) via stirring for 180 min at room temperature, and then alkali-activated FA (A-FA) particles were obtained via washing and drying.
2. Silane modification: Using deionized water as a solution, alkali-activated FA (A-FA) particles were dispersed in 1%, 2%, 4%, and 10% coupling agent solutions via stirring for 90 min at different temperatures (20, 40, 60 °C, respectively). Then, modified-FA particles (M-A-FA) were obtained by washing them 3 times and drying them at 80 °C for 24 h.

### 2.3. Characterization

FEI Nova Nano scanning electron microscope (SEM) 450 was used to analyze the surface morphologies of FA before and after modification. The variation in the surface chemistry of FA during the electroless Ag plating process was studied with the help of the Escalab250Xi (ThermoFisher Scientific, USA) X-ray photoelectron spectrometer (XPS). Also, the XPS was used to indicate whether the coupling agent was successfully coated on the FA surface. The surface wettability of FA and A-FA was studied using a surface tension meter (KRUSS K100). The degree of silane hydrolysis was monitored by a conductivity meter. A laser diffraction particle size Analyzer (LPSA (Malvern, UK), Mastersizer 2000E) was used to measure the particle size distribution of FA.

## 3. Results and Discussion

### 3.1. Morphologies and Particle Sizes of FA and A-FA Particles

**Figure 1:**
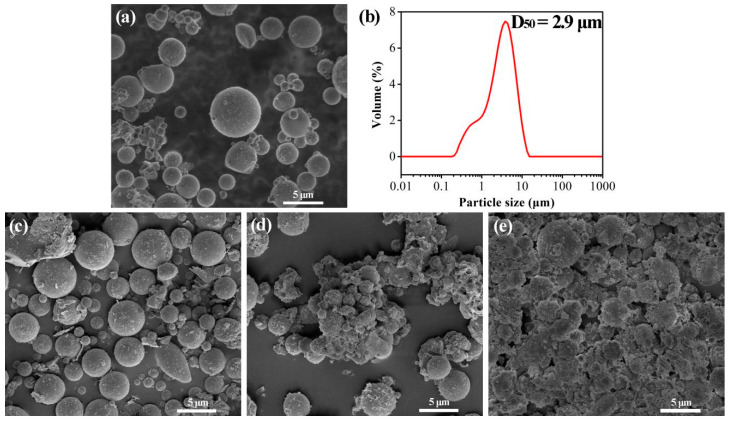
Morphologies and particle sizes of FA (SH012F) and A-FA particles: (**a**) SH012F, (**b**) particle size of SH012F, (**c**) 0.3-A-012FA, (**d**) 0.6-A-012FA, and (**e**) 1.2-A-012FA.

Based on previous research [14], FA particles were pretreated with different concentrations of the NaOH solution, and the morphologies of untreated FA and alkali-activated FA particles are shown in Figure 1. From Figure 1a, we can observe that the graded FA particles are mainly spherical with only a few irregular shapes, and the particle size distribution of FA was measured to be D_50_ = 2.9 μm, as shown in Figure 1b. It can be clearly seen that the particle size distribution of SH012F is narrow. Smaller particles stick together, attributed to its high surface activity, as shown in Figure 1a. The morphologies of alkali-activated FA particles with concentrations of 0.3, 0.6, and 1.2 mol/L of the NaOH solution are shown in Figure 1c–e, respectively. From Figure 1c, we can observe that there is no significant change in the FA surface after alkali activation with the 0.3 mol/L NaOH solution. However, the polymer reaction occurs on the FA surface after alkali activation in the 0.6 mol/L NaOH solution, resulting in the adhesion of FA particles, especially for small particles. The larger particles showed slight surface polymerization but did not result in cross-linking, which indicates that the smaller the particle size of FA, the higher the surface activity and the stronger the geopolymer reaction. It can be clearly seen that all FA particles react and crosslink together with an increase in the NaOH concentration, as shown in Figure 1e. In conclusion, the concentration of NaOH varies with the different particle sizes of FA. This is significant in the selection of a suitable concentration for alkali activation.

**Figure 2:**
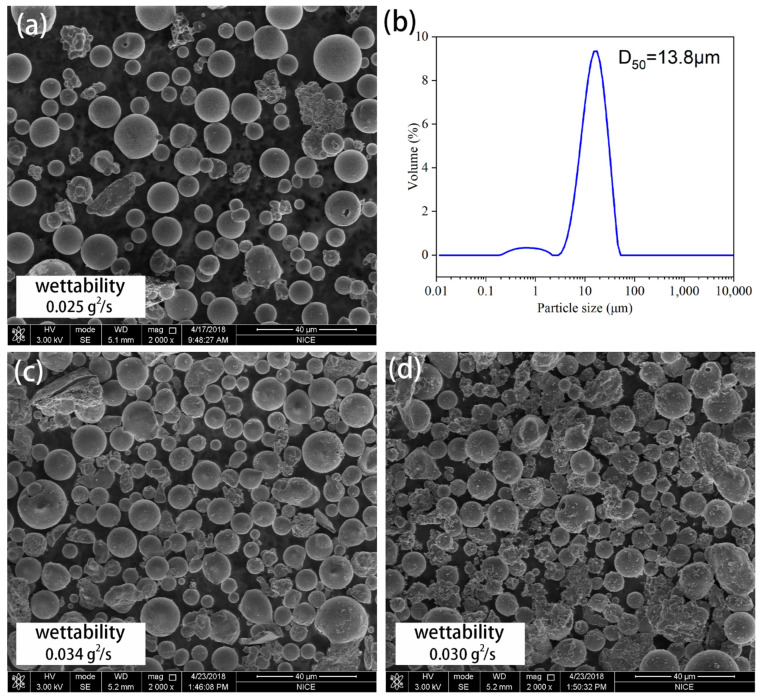
Morphologies and particle size of FA (SH090) and A-FA cenopheres: (**a**) SH090, (**b**) particle size, (**c**) 0.6-A-090FA, and (**d**) 1.2-A-090FA.

For large particle sizes of FA (SH090), the morphologies and particle sizes of FA and A-FA particles are shown in Figure 2. Compared with SH012F, untreated-FA (SH090) has a smooth surface and good sphericity, and its average particle size is 13.8 μm, as shown in Figure 2a,b. After classification, the particle size distribution of SH090 became narrow, which indicates that the particle size of graded FA is even. Also, fly ash particles are good for silane modification because of their larger size and better dispersion. Figure 2c shows the morphologies of alkali-activated FA with a concentration of 0.6 mol/L of NaOH. It can be clearly seen that there is no significant change in the surface of FA after alkali activation, with good dispersion and no cross-linking, which indicates that there is no geopolymer reaction occurring or the geopolymer reaction is not strong. In comparison with Figure 1e, it can be clearly observed that the surface of FA (SH090) treated with 1.2 mol/L NaOH showed a slight polymer reaction and a roughened surface without cross-linking, as shown in Figure 2d. This further indicated that it is not easy to cross-link NaOH with a large particle size of FA because of its low activity. Fly ash particles have good hydrophilicity because of the hydroxyl on their surfaces, so it is difficult to evaluate the surface wettability with the contact angle. A surface tension meter was used to evaluate the surface wettability of FA before and after alkali activation. The surface wettability of untreated FA was 0.025 g^2^/s. After alkali activation, the surface wettability of 0.6-A-090FA and 1.2-A-090FA was 0.034 g^2^/s and 0.030 g^2^/s, respectively. Compared with 0.6-A-090FA, the surface wettability of 1.2-A-090FA decreased, which was attributed to the corroding of the active substance on the FA surface by 1.2 mol/L NaOH. 0.6-A-090FA will be used for silane modification because of its high surface wettability as this high wettability is beneficial to silane modification.

In conclusion, 0.6-A-090FA (0.6-A-FA) will be chosen for the research on silane coupling agent modification and the mechanism between pretreated FA (alkali activation and silane modification) and silver particles.

### 3.2. Conductivity of Silane Solution

Silane coupling agents have been used recently as an environmentally friendly pre-treatment for FA, in order to achieve increased adhesion between silver films and the FA surface [15]. Usually, silanes used as adhesion promoters are applied as a water–alcohol solution [16,17]. At the very beginning of solution preparation, silane solutions undergo two reactions, namely, the hydrolysis of alkoxy groups and the condensation of silanol groups [18]. The functional groups of silane are responsible for the establishment of the covalent bridge between the attached mineral and the polymer chain [19,20]. The properties of the FA modified by silane coupling agents are influenced by the hydrolysis time, hydrolysis temperature, chemistry of silane molecules, and concentration of silane solution [21,22]. Therefore, a solution for determining the optimal hydrolysis time, hydrolysis temperature, and deionized water ratio in the silane solution can be easily obtained when the concentration of silanol reaches the maximum point by monitoring the conductivity of the silane solution over time [23].

**Figure 3:**
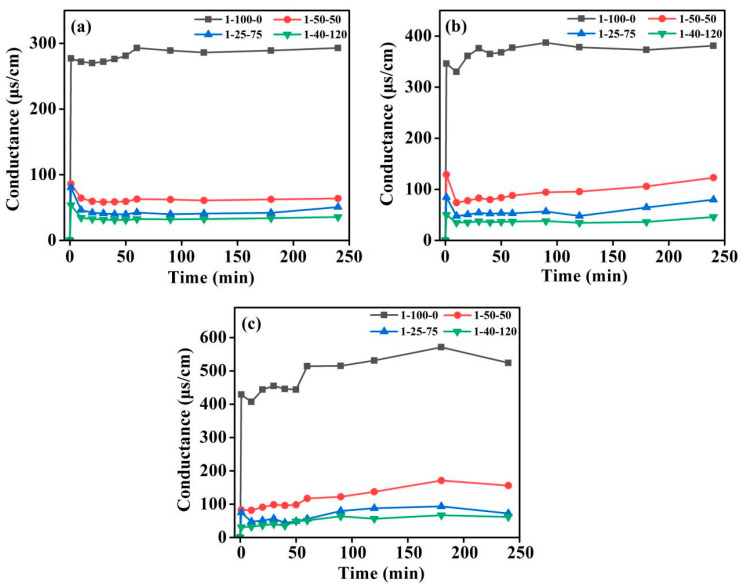
The variation in the conductivity of silane solutions: (**a**) 20 °C, (**b**) 40 °C, and (**c**) 60 °C.

The silane coupling agent (KH-792) was used as an example to explore the effects of hydrolysis temperature, hydrolysis time, and the ratio of deionized water on the conductivity of the hydrolyzed solution. Figure 3 shows the variation in the conductivity of the investigated silane solution versus hydrolysis time with an increase in the hydrolysis temperature and water fraction. One may see a similar trend of the variation in conductivity for the three solutions (#1:1-100-0, #2:1-50-50, and #3:1-25-75) at different hydrolysis temperatures, described by an increase in the conductivity in the first 1 min, followed by a plateau. As shown in Figure 3, solution #1, solution #2, and solution #3 refer to the ratios of silane coupling agent, deionized water, and methanol of 1:100:0, 1:50:50, and 1:25:75, which means the deionized water fraction decreases in these three solutions. It can be noted that the conductivity of solution #1 is much higher than that of solution #2 and solution #3 at different hydrolysis temperatures. It can be clearly seen that the conductivity of solution #1 increases, and no significant change in solutions #2 and #3 is observed with the increase in the hydrolysis temperature.

In conclusion, the optimal hydrolysis temperature of the silane solution should be 60 °C. Solution #1 has the highest conductivity due to the 100% deionized water and no methanol. In theory, a large amount of hydroxyl is exposed on the FA surface after alkali activation, so it can be easily combined with the silicon hydroxyl of the silane coupling agent to be uniformly coated on the A-FA surface. In order to ensure the silane coupling agent coats A-FA, the hydrolysis time was set to 90 min.

### 3.3. XPS Analysis of M-A-FA

Different kinds of silane coupling agents, including KH792, KH560, and MTMS, were selected to modify alkaline-activated FA (A-FA), and XPS was used to analyze whether the FA surface was successfully coated by the silane coupling agents. Our previous work proved that the alkali-activated FA was coated by the KH792 silane coupling agent [14]. For FA modified by KH792, two chemical states, 399.1 eV (-NH_2_) and 401.2 eV (-NH-), of N1s spectra were detected on the surface of FA, and the N/Si atomic ratio increased to 0.086, which was 4.5 times higher than that of pure FA, indicating that the FA surface was successfully coated with KH-792 [14]. Unsaturated functional groups ((-NH_2_) and (-NH-)) can adsorb silver ions and form the N–Ag coordination bond, increasing the bonding strength between the silver-plated layer and the FA surface and forming a uniform and compact silver film.

**Figure 4:**
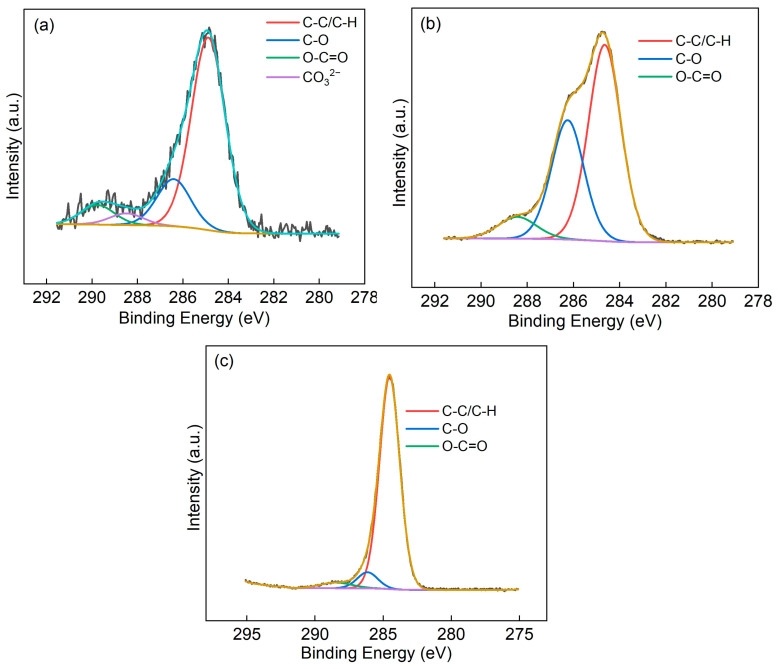
C1s spectrum of A-FA (**a**) and M-A-FA modified by KH560 (**b**) and MTMS (**c**).

Similarly, XPS analysis was carried out for A-FA before and after modification by KH560 and the C1s spectra were fitted, as shown in Figure 4. There are four clearly defined contributions in the C 1s spectra assigned; in the order of increasing binding energy (BE), they are C-C/C-H, C-O, O-C=O, and C bonded to three O atoms [24,25]. The shifts relative to the hydrocarbon peak are 1.51, 3.83, and 4.85 eV for the C-O, O-C=O, and CO_3_^2−^ components, respectively [26], which are present on all surfaces exposed to the air and carbonate of FA, as shown in Figure 4a.

The atomic percentages of elements in A-FA and M-A-FA are shown in Table 2. It can be seen clearly that the atomic percentage of C/Si was 0.37 before modification with the silane coupling agent. After the modification by KH560, it was found that the C/Si atomic ratio increased to 3.09, indicating that the FA surface was successfully coated with KH560. Also, there are three clearly defined contributions in the C 1s spectra assigned; in the order of increasing BE, they are C-C/C-H, C-O, O-C=O, and the C-O group, corresponding to the ether bond and epoxy bond in KH560 [27,28,29]. The atomic ratio between the C-O group and Si increased from 0.06 to 1.08, increasing by 18 times after KH-560 modification, indicating that A-FA surfaces were successfully coated with KH560. Using quantitative analysis of A-FA surfaces modified by MTMS, it was found that the C/Si atomic ratio increased from 0.37 to 1.31, indicating that the FA surfaces were coated with MTMS. The split-peak fitting of C1s showed that C-C/C-H, C-O, and O-C=O also existed [30], and the atomic ratio of C-C/C-H group to Si increased from 0.26 to 1.16, increasing by 4.46 times after modification with MTMS, indicating that A-FA surfaces were successfully coated with MTMS.

In summary, the FA surfaces after alkaline cleaning were successfully coated with these three silane coupling agents, and it was found that under the same coating condition, the coated amount of KH560 was the highest, KH792 was the second, and MTMS was the lowest.

### 3.4. Utilization of M-A-FA and Its Function in Silane Group

**Figure 5:**
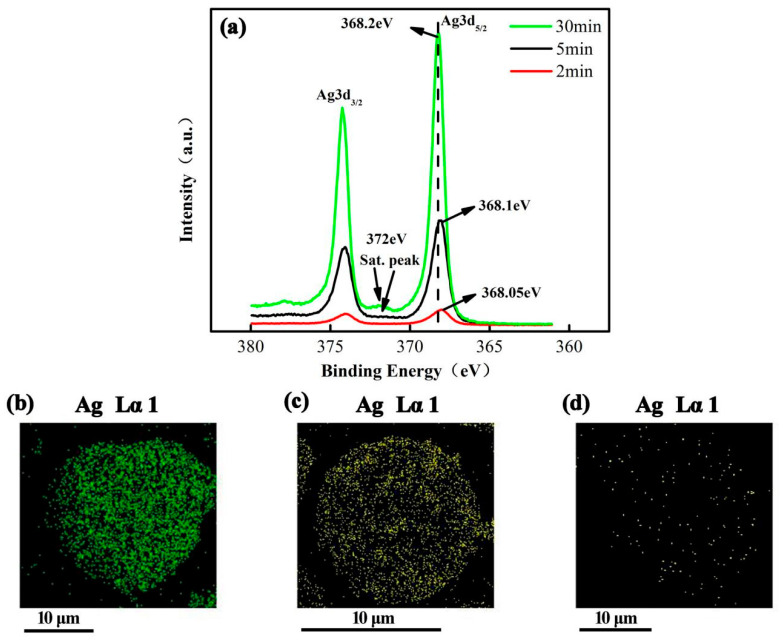
High-resolution XPS spectra and EDS mapping: (**a**) XPS spectra of Ag3d for 0.6-FA-S-Ag, (**b**) EDS mapping of Ag particles for 0.6-FA-S-Ag-30 min, (**c**) EDS mapping of Ag particles for 0.6-FA-S-Ag-5 min, and (**d**) EDS mapping of Ag particles for 0.6-FA-S-Ag-2 min.

Our previous work proved that the N-Ag coordination bond of Ag-plated M-A-FA modified by KH792 was detected, resulting in strong bond strength between the silver layer and the FA surface. Our previous work also demonstrated that XPS is a good surface analysis instrument with a detection depth of less than 10 nm. When Ag-plated FA conditions were changed to reduce the thickness of the silver plating layer on the FA surface, a bond between N and Ag could be easily detected by XPS, as shown in Figure 5. Figure 5a shows the XPS spectra of Ag3d for 0.6-FA-S-Ag after silver plating for 30 min, 5 min, and 2 min, respectively. Figure 5b–d shows the EDS mapping of Ag particles plated on the FA surface, corresponding to Figure 5a. In Figure 5a, it can be clearly seen that satellite peaks near 372 eV were observed after plating for 30 min and 5 min, attributed to the metallic silver, which is consistent with the EDS mapping in Figure 5b,c. In order to detect the shift in the Ag3d peak, the silver plating time was further reduced to 2 min, and Ag-plated FA (0.6-FA-S-2 min) with a small amount of Ag particles was obtained, as shown in Figure 5d. The XPS spectra of Ag3d for 0.6-FA-S-2 min is shown in Figure 5a and no satellite peak was observed. Meanwhile, compared with the peak of elemental silver, the peak of Ag3d for 0.6-FA-S-2 min shifted from 368.2 eV to 368.05 eV, illustrating that the coordination bonding of N-Ag did occur. In conclusion, the chemical bond between the silver layer and the silane coupling agent on the FA surface can be detected by reducing the silver particles plated on the FA surface. Similarly, when exploring the existence of chemical bonds of KH560 and MTMS between the FA surface and the silver layer, the same method was used to reduce the thickness of the silver layer by reducing the concentration of the AgNO_3_ solution and the reaction time of silver plating.

**Figure 6:**
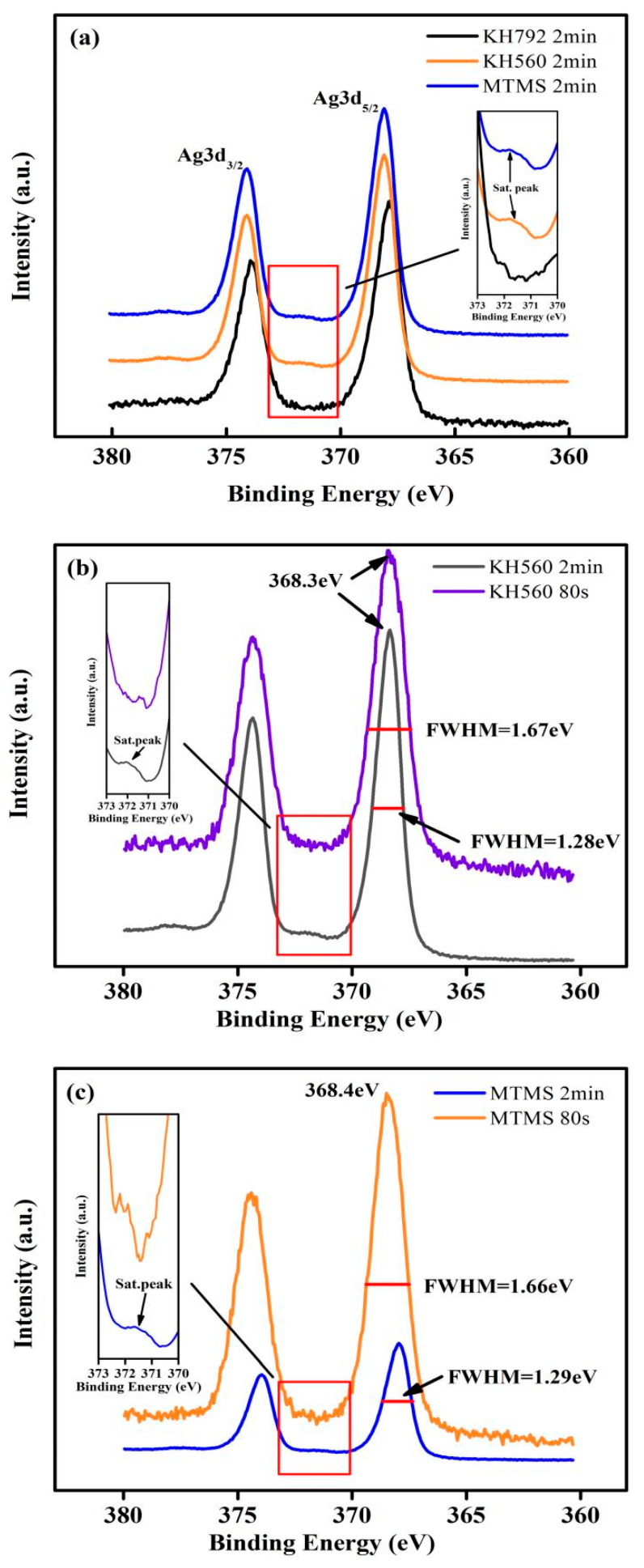
(**a**) Ag3d spectrum of Ag-plated M-A-FA modified by KH792, KH560, and MTMS after plating for 2 min; (**b**) Ag3d spectrum of Ag-plated M-A-FA modified by KH560 after plating for 2 min and 80 s; (**c**) Ag3d spectrum of Ag-plated M-A-FA modified by MTMS after plating for 2 min and 80 s.

Under the same conditions regarding the plating solution concentration and plating time (2 min), it can be seen in Figure 6a that the FWHM of the Ag3d spectrum modified by KH560 and MTMS was equivalent to that of the one modified by KH792, while the satellite peak of Ag element could still be seen. It is difficult to detect the chemical bond between the silane coupling agent and the silver layer when the satellite peak of Ag exists. When the plating time was reduced to 80 s, the FWHM of the Ag3d spectrum became wider, and the satellite peak of Ag disappeared, as shown in Figure 6b,c. It can be seen clearly that the FWHM of the Ag3d spectrum modified by KH560 and MTMS increased from 1.28 eV to 1.67 eV and 1.29 eV to 1.66 eV after shortening the plating time to 80 s, respectively. The reason was likely that the size of the silver layer on the FA surface decreased, so the signal-to-noise ratio of the spectrum decreased instead of changing the silver’s chemical state. For the silver-coated samples, we used Al2p 74.4 eV as a reference for charge correction, which would not change its chemical state before and after silver plating. It was found that when the silver plating time was 80 s, the Ag3d5 peaks of the silver-coated FA modified by KH560 and MTMS were 368.3 eV and 368.4 eV, which were classified as Ag. After KH560 and MTMS modification, the Ag chemical states of silver-coated FA were only in three forms: Ag, Ag_2_O, or AgO. However, the binding energy of Ag3d5 peaks for Ag_2_O and AgO were lower than that of Ag, which were 367.7 ± 0.2 eV and 367.4 ± 0.2 eV, respectively [31,32]. It could be determined that although FA surfaces were modified by KH560 and MTMS, there were no chemical bonds generated during silver plating, and the silver layers existed as Ag.

**Figure 7:**
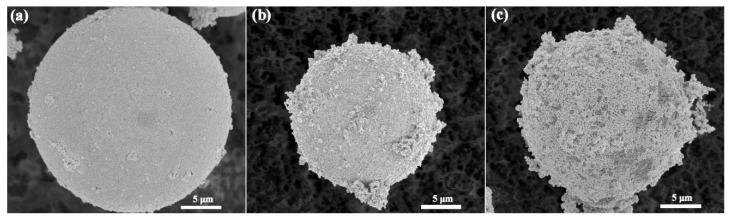
Morphologies of Ag-plated M-A-FA modified by three kinds of coupling agents: (**a**) 0.6-FA-S-Ag-KH792, (**b**) 0.6-FA-S-Ag-KH560, and (**c**) 0.6-FA-S-Ag-MTMS.

The SEM images showed that the silver layers on FA surfaces modified with KH792 were uniform and there was no free silver element observed on the FA surface, as shown in Figure 7a. However, the silver layers on FA surfaces modified by KH560 were rough and the free element silver agglomerated, as shown in Figure 7b. It can be seen clearly that more FA surface was exposed, and the agglomeration of the free silver element was more serious after modification with MTMS, as shown in Figure 7c. The above SEM results are consistent with the XPS results. The silver layers on FA surfaces modified by KH792 were uniform with no changes or cracks after ultrasonic oscillation and cutting, which indicated that the adhesive strength between the Ag film and FA modified by KH-792 was strong enough [14]. After modification with KH560 and MTMS, the silane coupling agents on the FA surface and Ag particles had no chemical bonds, the Ag particles only accumulated on the FA surface, and the uniformity of the silver layers was poorer. More agglomerations caused barer (uncoated) surface spots. The amount of silane coupling agent (KH560) on the FA surface was much more than that modified by MTMS, so the silver layers on the FA surface modified by KH560 were more uniform than those modified by MTMS.

## 4. Conclusions

The activation effect of FA was related to the concentration of the NaOH solution and FA particle sizes. The results showed that the surface wettability of smaller FA particles sized 13.8 μm increased from 0.025 g^2^/s to 0.034 g^2^/s after activation via a 0.6 mol/L NaOH solution for 180 min, which is suitable for silane modification and electroless plating. The surface hydrophilicity of FA improved after activation with the NaOH solution, and the optimal hydrolysis temperature and time of silane solution are 60 °C and 90 min, respectively. The XPS results indicated that KH-560 and MTMS were successfully coated on FA surfaces. With the same silver plating conditions, there were still satellite peaks observed after silver plating for 2 min for M-A-FA modified by KH560 and MTMS. It was found that the satellite peak of Ag disappeared after reducing the plating time to 80 s, and the Ag3d5 peaks of the silver-plated FA modified by KH560 and MTMS were 368.3 eV and 368.4 eV, which was classified as Ag, indicating that there were no chemical bonds between silver layers and FA surfaces. In addition, the SEM showed that Ag particles agglomerated on FA surfaces, the uniformity of the silver layers was poor, and even bare surfaces were found after modification with KH560 and MTMS, which further proved the lack of chemical bonds between the silver layers and the silane coupling agents on FA surfaces. The silver layers on FA surfaces modified with KH792 were uniform and there was no free silver element observed, which means that the appropriate silane coupling agent can improve the distribution of silver particles on FA surfaces. Thus, XPS was used to analyze whether silane coupling agents can be coated on FA surfaces and even to detect whether the chemical bonds between silver layers and silane coupling agents are generated by shortening the silver plating time so that the satellite peak disappears.

## Figures and Tables

**Table 1:** The chemical compositions and combustion loss of FA.

XRF	SiO_2_	Al_2_O_3_	CaO	Fe_2_O_3_	TiO_2_	K_2_O	SO_3_	Na_2_O	MgO	Firing Loss
SH012F	41.94	28.63	8.86	5.75	1.32	1.19	0.98	0.85	0.57	3.40%
SH090	43.57	30.2	5.9	4.5	1.35	1.21	0.18	0.84	0.59	6.90%

**Table 2:** Atomic percentages of elements in A-FA and M-A-FA.

Samples	C (%)	C-C (%)	C-O (%)	O=C-O (%)	CO_3_^2−^ (%)	Si (%)	C/Si	C-O/Si	C-C/Si
A-FA	26.91	19.14	4.66	1.17	1.94	73.09	0.37	0.06	0.26
M-A-FA (KH560)	75.51	43.41	26.39	5.71	\	24.49	3.09	1.08	\
M-A-FA (MTMS)	56.74	50.66	3.91	2.17	\	43.26	1.31	\	1.16

## Data Availability

The original contributions presented in the study are included in the article, further inquiries can be directed to the corresponding author.
